# Interactions of the Lysosomotropic Detergent O-Methyl-Serine Dodecylamide Hydrochloride (MSDH) with Lipid Bilayer Membranes—Implications for Cell Toxicity

**DOI:** 10.3390/ijms21093136

**Published:** 2020-04-29

**Authors:** Ana-Maria Villamil Giraldo, Ida Eriksson, Stefan Wennmalm, Timmy Fyrner, Thomas Ederth, Karin Öllinger

**Affiliations:** 1 Experimental Pathology, Department of Biomedical and Clinical Sciences, Linköping University, SE-581 85 Linköping, Sweden; ana.villamil@icm.uu.se (A.-M.V.G.); ida.eriksson@liu.se (I.E.); 2 Royal Institute of Technology, Department of Applied Physics, Biophysics, SciLifeLab, 171 65 Solna, Sweden; stewen@kth.se; 3 Division of Chemistry, IFM, Department of Physics, Chemistry and Biology, Linköping University, SE-581 83 Linköping, Sweden; timmy.fyrner@northwestern.edu; 4 Division of Biophysics and Bioengineering, IFM, Department of Physics, Chemistry and Biology, Linköping University, SE-581 83 Linköping, Sweden; thomas.ederth@liu.se

**Keywords:** MSDH, lysosome, lysosomotropic detergent, lysosomal membrane permeabilization, liposome

## Abstract

O-methyl-serine dodecylamine hydrochloride (MSDH) is a detergent that accumulates selectively in lysosomes, a so-called lysosomotropic detergent, with unexpected chemical properties. At physiological pH, it spontaneously forms vesicles, which disassemble into small aggregates (probably micelles) below pH 6.4. In this study, we characterize the interaction between MSDH and liposomes at different pH and correlate the findings to toxicity in human fibroblasts. We find that the effect of MSDH on lipid membranes is highly pH-dependent. At neutral pH, the partitioning of MSDH into the liposome membrane is immediate and causes the leakage of small fluorophores, unless the ratio between MSDH and lipids is kept low. At pH 5, the partitioning of MSDH into the membrane is kinetically impeded since MSDH is charged and a high ratio between MSDH and the lipids is required to permeabilize the membrane. When transferred to cell culture conditions, the ratio between MSDH and plasma membrane lipids must therefore be low, at physiological pH, to maintain plasma membrane integrity. Transmission electron microscopy suggests that MSDH vesicles are taken up by endocytosis. As the pH of the endosomal compartment progressively drops, MSDH vesicles disassemble, leading to a high concentration of increasingly charged MSDH in small aggregates inside the lysosomes. At sufficiently high MSDH concentrations, the lysosome is permeabilized, the proteolytic content released to the cytosol and apoptotic cell death is induced.

## 1. Introduction

The lysosomes are acidic organelles involved in several central processes in the cell including degradation, metabolic homeostasis, and cell death [1,2]. Upon lysosomal membrane permeabilization (LMP), the hazardous hydrolytic content of the lysosome is released to the cytosol. Several earlier publications have shown that the extent of membrane permeabilization determines the fate of the cell. Massive lysosomal rupture causing cytoplasmic acidification and uncontrolled degradation of cellular constituents might lead to necrosis. However, partial and selective LMP causes different forms of regulated cell death [3,4,5]. Following LMP, the downstream signaling involves classical apoptosis induced through caspase activation by the intrinsic or extrinsic pathways [6,7,8], or caspase-independent death with apoptosis-like morphology [9]. Lysosomal stability is affected by a plethora of factors and, although several compounds have been identified as both stimulating and preventing the loss of lysosomal membrane integrity, no general mechanism of LMP has been identified [8,10].

The ability of lysosomes to induce cell death signaling makes them interesting targets in cancer therapy, particularly for apoptosis- and multidrug-resistant cancers [11,12]. In particular, weak bases are attractive candidate molecules since they accumulate in the acidic milieu of the lysosomes, become protonated and thereby are prevented from diffusing back into the cytosol. This allows for up to 1000-fold drug accumulation inside the acidic organelles [13].

Detergents are amphiphilic molecules, where the poor solubility of their non-polar parts induce aggregation in aqueous solutions. This occurs at a specific concentration (in general, the Critical Aggregation Concentration (CAC) or, if the aggregation is in the form of micelles, the Critical Micelle Concentration (CMC)), which depends on the structure of the molecule, and the solution properties. Detergents are useful molecular tools in biophysics and molecular biology, where common uses include the solubilization and reconstitution of membrane proteins [14], gene [15] and drug [16] delivery, as well as for membrane solubilization [17]. Great attention has been paid to lipid membrane solubilization by detergents [17,18,19], but the formulation of general principles for the action of detergents on lipid membranes is difficult due to the specific effects of detergent structure on the interaction [20,21,22]. Lysosomotropic detergents (LDs) have, beside the basic amine group common for many lysosomotropic agents, a long hydrophobic chain which makes them surface active, resulting in detergent properties. Since the publication of an early paper on LDs by Firestone and Pisano [23], it has been widely assumed in the subsequent literature, probably based on a statement in their paper, that such LDs acquire detergent-like properties only in the acidic interior within the lysosomes, rather than having surface activity on the plasma membrane [23]. Specifically, upon accumulation inside the lysosome, the hydrophobic chain of the LD is buried in the membrane and when the concentration is sufficiently high, the lysosomal membrane will become permeabilized [24]. O-methyl-serine dodecylamine hydrochloride (MSDH), belonging to the LDs, has an amine group with pK_a_ of 5.9 and a hydrophobic chain [24]. Evidence of lysosomal-dependent cell death after MSDH exposure has been obtained from several different cell systems [4,25]. Although destabilization of the lysosomes has been established, the mechanism(s) by which LDs enter cells and rupture lysosomal membranes need further investigation. In a recent study detailing the properties of MSDH, we found that MSDH spontaneously formed vesicles (50–500 nm in diameter) at cytosolic pH, whereas at pH < 6.4 the vesicles disassembled into very small aggregates, most likely micelles [26]. This study also found a CAC of approximately 10 µM. These results, in conjunction with the rapid uptake of MSDH in lysosomes, are not in accordance with the commonly held view that MSDH, at physiological pH, remains in the form of monomers in solution [14] and enters cells as monomers via diffusion over the membranes [24]. First, since the MSDH vesicles are in equilibrium with free monomers at the CAC, the low abundance of free molecules, which would also partition into any other lipid structure available in the cell, implies that the diffusion of monomers into lysosomes is a slow process. Second, MSDH aggregates are positively charged at cytosolic pH [26], and cationic liposomes have been shown to traverse plasma membranes rapidly via endocytotic pathways [27]. These observations rather suggest an endocytic uptake mechanism.

Endocytosis is a complex mechanism and several specific and intermixed mechanisms are defined, including clathrin-dependent and independent mechanisms and pinocytosis/fluid phase mediated uptake [28]. Internalization via endocytosis is an important signaling pathway, e.g., at the synaptic end where signals often occur in an ultrafast (<0.1 s) or fast (1–10 s) manner in order to recycle exocytosed vesicles [29]. These events are usually clathrin-independent, while clathrin-mediated endocytosis is considered a more general “housekeeping” process that requires the assembly of clathrin-coated pits, and thus occurs somewhat slower (10–50 s) [30]. The established lysosomotropic agent L-leucyl-L-leucine methyl ester (LLOMe) is taken up by receptor-mediated endocytosis and gains membranolytic properties by the action of the lysosomal enzyme dipeptidyl peptidase-I inside the lysosome [31,32]. Studies have shown that LLOMe induces lysosomal damage within minutes after addition [33,34], demonstrating that delivery to lysosomes via endocytosis is a rapid process.

In the present study, we investigate the stability of lysosome-like liposomes exposed to MSDH and characterize their stability at different pH values. These findings were transferred to cell culture models, where we could correlate the mechanisms of MSDH-induced LMP to cell toxicity and investigate the mode of entry through the plasma membrane.

## 2. Results

### 2.1. MSDH-induced Leakage of Small and Large Molecules is Affected by pH and Lipid Composition

**Figure 1:**
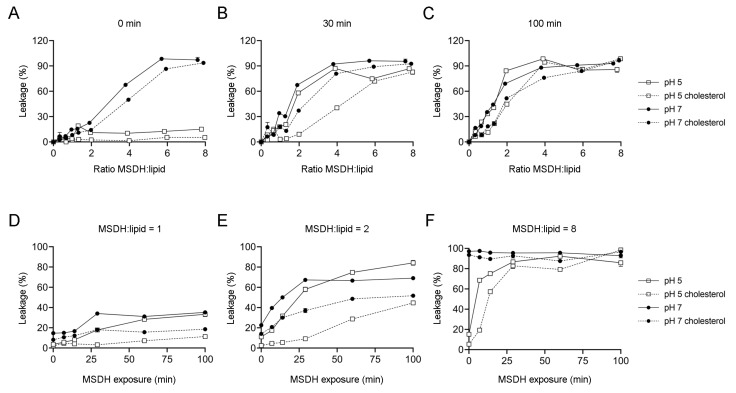
Leakage of small molecules from liposomes. The dye/quencher pair 8-aminonapthalene-1,3,6 trisulfonic acid/p-xylene-bis-pyridinium bromide (ANTS/DPX) (350 Da) was encapsulated in liposomes containing 40% or no cholesterol. Liposomes were mixed with increasing concentrations of O-methyl-serine dodecylamine hydrochloride (MSDH) and ANTS leakage was analyzed (**A**) immediately, (**B**) 30 min and (**C**) 100 min after adding MSDH at pH 7 or pH 5. Analysis of time dependent leakage of ANTS from liposomes with estimated MSDH/lipid ratios of (**D**) one, (**E**) two and (**F**) eight. In total, 100% leakage corresponds to samples exposed to Triton X-100. The results are shown as mean ± SD, *n* > 3. Most errors are smaller than the size of the symbols.

To characterize the MSDH-induced leakage of small molecules from lipid vesicles, we used the dye/quencher pair 8-aminonapthalene-1,3,6 trisulfonic acid/p-xylene-bis-pyridinium bromide (ANTS/DPX). The molecular weight of these molecules is around 350 Da, which would be the equivalent to a three-residue peptide. ANTS and DPX are encapsulated into liposomes under conditions in which ANTS fluorescence is quenched and the increase in its fluorescence emission is the result of leakage into the larger extravesicular volume. Leakage measurements were performed either at pH 5 or 7 to mimic the conditions at the lysosome and plasma membranes, respectively. The release of fluorophore was analyzed at MSDH/lipid ratios between zero and eight. Immediately after the addition of MSDH, no leakage was observed at pH 5 for any MSDH/lipid ratio tested, while at pH 7, leakage was apparent already at MSDH/lipid ~2. However, leakage was reduced in liposomes containing 40% cholesterol (Figure 1A). This large difference in the extent of leakage between liposomes incubated with MSDH at pH 5 or at pH 7 was less evident after 30 min (Figure 1B), and was eliminated after 100 min. Despite this, the difference observed in the presence of cholesterol was evident, especially at MSDH/lipid ratios ≤2 (Figure 1C). The time-dependence of the fluorophore leakage induced by MSDH at MSDH/lipid ratios of one, two and eight is presented in Figure 1D–F.

**Figure 2:**
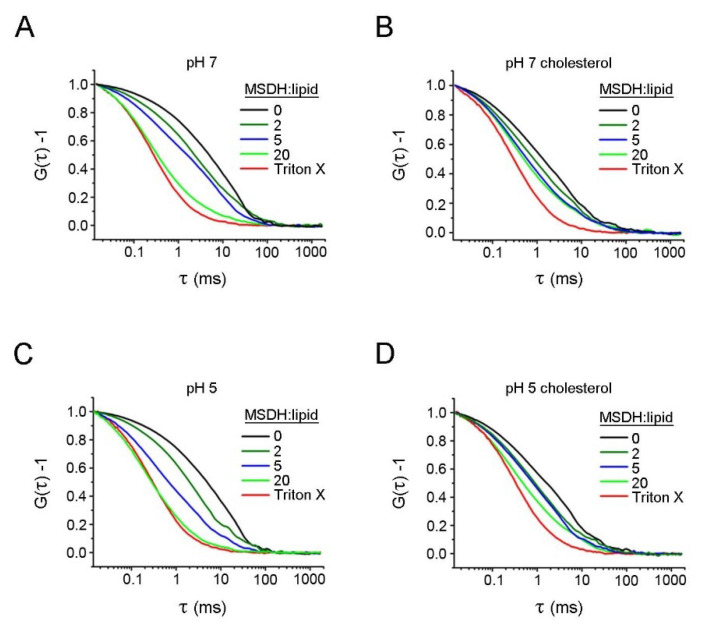
Fluorescence correlation spectroscopy (FCS) analysis of MSDH-induced leakage of large molecules from liposomes. Dextran–rhodamine (TRITC-dextran) (40 kDa) was encapsulated in liposomes containing 40% or no cholesterol. Liposomes were mixed with increasing concentrations of MSDH and leakage was analyzed using fluorescence correlation spectroscopy (FCS). Triton X-100 was used to completely disintegrate the liposomes. Normalized autocorrelation data from FCS measurements in (**A**) liposomes at pH 7 prepared without or (**B**) containing 40% cholesterol. (**C**) Liposomes at pH 5 prepared without or (**D**) containing 40% cholesterol. Each curve is the average of >3 measurements of 20 s each.

To gain insight into the process of leakage of larger molecules, we used liposomes loaded with 40 kDa dextran–rhodamine (TRITC-dextran 40) and followed the release of the dextran by fluorescence correlation spectroscopy (FCS). FCS measures the diffusion of the slower dextran-containing vesicles as well as the faster, free dextran molecules. Under the conditions tested (pH 7, pH 5, liposomes with or without 40% cholesterol), the extent of leakage increased with the ratio of MSDH/lipids (Figure 2A–D). At an MSDH/lipid ratio of 20, the mean diffusion rates in samples without cholesterol were almost as fast as in the presence of Triton X-100, 5 min after the addition of MSDH. Under identical conditions, vesicles containing 40% cholesterol exhibited diffusion times slightly higher than those containing no cholesterol, meaning that more intact vesicles were still present (Table 1).

**Figure 3:**
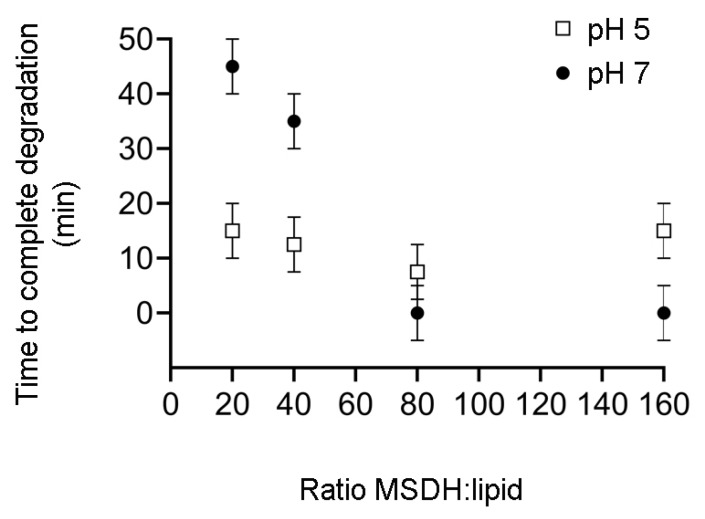
Time-dependence of complete degradation of liposomes. TRITC-dextran (40 kDa) was encapsulated in liposomes and mixed with increasing concentrations of MSDH. Leakage was analyzed at pH 7 and at pH 5 using fluorescence correlation spectroscopy. Degradation was considered complete when no spikes above 1500 kHz were visible during the entire measurement. For each data point, a series of FCS measurements was recorded for 40 min. The data is presented as the mean ± SD, estimated from multiple independent (*n* > 3) measurements. *p* < 0.05 for MSDH/lipid ratio 40 vs. 80 and 20 vs. 80 at pH 7.

In the FCS measurements presented so far, though the mean diffusion time at a 20:1 MSDH/lipid ratio approached that when Triton X-100 was used, intact vesicles were still present. We therefore investigated the time until complete degradation, defined as a measurement with no or only a single small spike, corresponding to a transiting liposome, visible in the intensity trace. Here, the MSDH/lipid ratios ranged from 20 to 160, with the lipid concentration kept constant at 50 µM. (Figure 3). At MSDH/lipid ratios between 20 and 80, complete degradation was time-dependent at pH 7, while no dependence was found at pH 5, where no vesicles were present 15 min after the addition of MSDH.

### 2.2. MSDH Causes Permeabilization of Cellular Membranes and Cell Death

**Figure 4:**
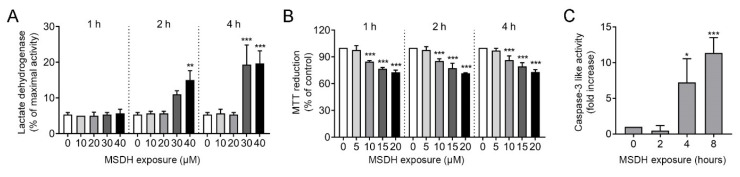
Concentration-dependent toxicity of MSDH. Fibroblasts were exposed to MSDH and toxicity was analyzed using (**A**) LDH activity in conditioned media after 1, 2 and 4 h of MSDH exposure (maximal LDH activity was obtained by total cell lysis), (**B**) cell viability using the 3-(4,5-dimethylthiazol-2-yl)-2,5-diphenyltetrazolium bromide (MTT) assay after 1, 2 and 4 h of MSDH exposure and, (**C**) caspase-3 like activity (15 µM MSDH). The results are shown as mean ± SD (*n* = 3) * *p* < 0.05 and *** *p* < 0.001 compared to untreated control at each time point.

The effect of MSDH treatment was then studied in human fibroblasts. Starting with a large concentration span, we investigated the concentration dependence of MSDH for plasma membrane lysis. By measuring the LDH activity in conditioned media (i.e., the media collected from MSDH-exposed cells), the amount of plasma membrane damage could be estimated. The addition of 10–40 µM MSDH showed that the plasma membrane was intact at concentrations ≤20 µM (Figure 4A), while concentrations ≥30 µM caused substantial and rapid plasma membrane damage and are therefore not suitable for experiments in fibroblasts. Thus, to avoid leakage over the plasma membrane, the cell culture experiments were performed at concentrations ≤20 µM. An analysis of cell viability, detected as a reduction in 3-(4,5-dimethylthiazol-2-yl)-2,5-diphenyltetrazolium bromide (MTT), showed that MSDH treatment caused a concentration-dependent loss of viability at concentrations ≥ 10 µM. The cell death never exceeded 50% with the tested concentrations (Figure 4B). To determine the cell death mechanism, caspase-3 activation was analyzed after treatment with 15 µM MSDH. A time-dependent increase in caspase-3 activity was found, indicating activation of the apoptosis pathway (Figure 4C).

**Figure 5:**
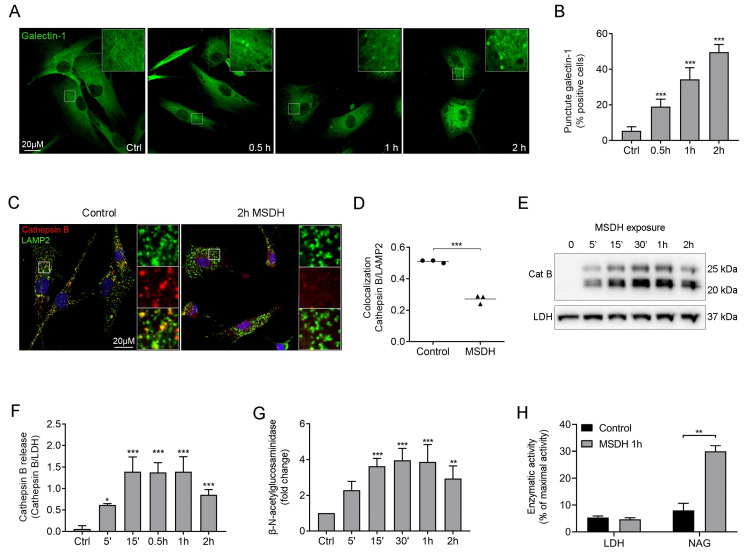
Lysosomal membrane permeabilization in response to MSDH treatment. Human fibroblasts were exposed to MSDH for 5 min–2 h. (**A**) Lysosomal rupture detected by Galectin-1 staining (green) in cells exposed to 15 µM MSDH, with (**B**) corresponding image analysis of Galectin-1 puncta-positive cells. (**C**) Immunostaining of cathepsin B (red), lysosomal-associated membrane protein-2 (LAMP2) (green) and nuclei (DAPI; blue) in cells exposed to MSDH (15 µM, 2 h). Merged image shows colocalization between cathepsin B and LAMP2 in yellow. (**D**) The estimated colocalization was obtained from image analysis in C. (**E**) Lysotracker staining of live cells before and after MSDH exposure (15 µM, 10 min). (**F**) Immunoblotting of active cathepsin B and lactate dehydrogenase (LDH) in cytosolic fractions from cells exposed to 15 µM MSDH, with corresponding densitometric analysis of cathepsin B release, correlated to LDH. (**G**) β-N-acetyl-glucoseaminidase (NAG) activity in cytosolic fractions. (**H**) Release of LDH and NAG to cytosol in cells exposed to 15 µM MSDH. The results are shown as mean ± SD (*n* = 3), * *p* < 0.05, ** *p* < 0.01 and *** *p* < 0.001 compared to untreated control.

The toxic properties of LDs, when added to cells at low concentrations, are initiated through mainly lysosomal-specific reactions, since the LDs are accumulated within lysosomes due to protonation in the acidic environment. As the concentration increases, lysosomal membrane stability is compromised [24]. Using the sensitive marker of lysosomal membrane permeabilization, galectin-1 [35], we found that 15 µM MSDH induced the translocation of galectin-1 from the cytosol (diffuse staining) to the lysosomes (puncta) indicating damage to the endo-lysosomal membranes (Figure 5A). Image analysis showed a time-dependent increase in the number of puncta-positive cells (Figure 5B). In accordance, the release of cathepsin B from the lysosomal lumen to the cytosol was detected by immunocytochemical staining (Figure 5C), and quantified by estimating co-localization between cathepsin B and lysosomal-associated membrane protein-2 (LAMP2) (Figure 5D). Reduced colocalization indicates the release of cathepsin B from the lysosome to the cytosol. Also, the number of Lysotracker-positive lysosomes was substantially reduced already after 10 min of MSDH exposure, demonstrating the loss of the proton gradient in the damaged lysosomes (Figure 5E). Immunoblotting of cathepsin B in cytosolic fractions showed that MSDH caused an almost instant injury to the lysosomal membrane, as cathepsin B was released within 5 min after MSDH exposure (Figure 5F). This was confirmed by a concomitant increase in cytosolic activity of the lysosomal hydrolase β-N-acetyl-glucosaminidase (NAG). The enzymatic activity was significantly increased in the cytosol 15 min after MSDH addition (Figure 5G) and reached its maximum activity within 30 min. By combining the results of LDH activity (Figure 4A) and NAG activity, we obtained a suitable concentration and exposure time for MSDH; 15 µM for 1 h, where we achieved significant LMP and no plasma membrane damage (Figure 5H).

Taken together, parallels can be established by comparing results from MSDH leakage analysis and cell toxicity experiments. At low ratios of MSDH/lipids at pH 7, the presence of cholesterol prevents the leakage of small molecules even after long incubation times (Figure 1), which matches the stability of the plasma membrane observed in our cell culture experiments (Figure 4A). At higher MSDH/lipid ratios, membrane solubilization occurs instantly at pH 7 and this is most probably the condition under which we observe the severe damage of the plasma membrane and acute cell death. Considerably higher ratios of MSDH are required at pH 5 in order to observe an equivalent extent of leakage (Figure 1). This corresponds to our observations that the plasma membrane is intact, while accumulation of MSDH inside the lysosome will eventually cause lysosomal leakage (Figure 5H).

### 2.3. Uptake of MSDH by Endocytosis

**Figure 6:**
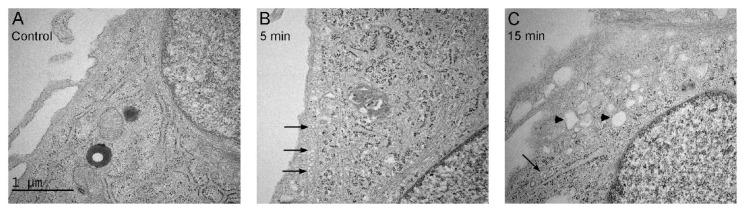
Enhanced endocytosis after exposure of fibroblasts to MSDH. Electron micrograph of fibroblasts exposed to 15 µM MSDH; (**A**) control (**B**) 5 min exposure, and (**C**) 15 min exposure. Arrows indicate small well-defined vacuoles with protein coat at the edge of the cell and arrowheads indicate larger vacuoles without protein coat. Magnification 40,000× and scale bar 1 µm.

Previously, we have shown that MSDH forms vesicles at pH 7 and we therefore speculated that MSDH enters cells by endocytosis [26]. Studies of the ultrastructure of fibroblasts revealed an increased number of small vacuoles in the close vicinity of the plasma membrane, 5 min after the addition of MSDH. An indication of a protein coating at the membrane suggests the activation of endocytosis. After 15 min, larger membrane-surrounded structures with no apparent protein coat were detected (Figure 6A–C).

**Figure 7:**
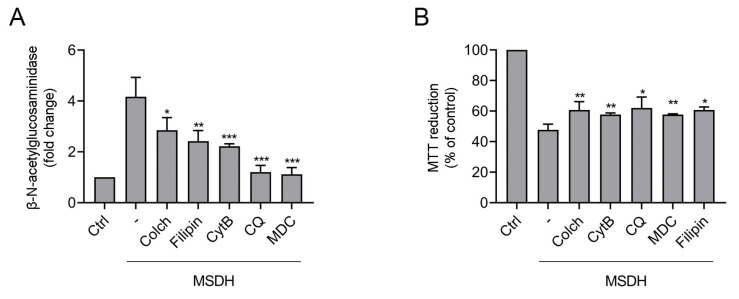
Effect of endocytosis inhibitors on MSDH toxicity. Endocytosis was prevented by pretreatment with chloroquine (CQ, 10 µM, 30 min), colchincine (Colch, 10 µM, 1 h), cytochalasin B (Cyt B, 5 µM, 1 h), mono-dansylcadaverine (MDC, 50 µM, 30 min) or filipin (0.5 µM, 30 min). (**A**) β-N-acetyl-glucoseaminidase (NAG) activity in cytosolic fractions after 30 min of MSDH exposure (15 µM). (**B**) Viability after 1 h of MSDH exposure (15 µM). Each inhibitor is compared with its respective control. Results are shown as mean ± SD, (*n* = 3), * *p* < 0.05, ** *p* < 0.01 and *** *p* < 0.001 compared to sample exposed to MSDH only.

Next, we determined if endocytosis inhibitors had any effect on lysosomal membrane integrity and cell viability. The addition of endocytosis inhibitors, mechanistically targeting different forms of endocytosis [36], revealed protection against MSDH-induced lysosomal membrane disruption (Figure 7A). The best protection was found for mono-dansylcadaverine (MDC), an inhibitor of clathrin-mediated endocytosis (CME), and chloroquine, which increases lysosomal pH and affects the function of clathrin and clathrin-coated vesicles [37]. Likewise, the microtubule destabilizing drug colchicine and the actin polymerization inhibitor cytochalasin B exhibited significant protection. The cholesterol-binding drug filipin also reduced MSDH-induced LMP. Filipin binds to and sequesters free cholesterol in the plasma membrane, a prerequisite for the internalization of several plasma membrane proteins through clathrin-independent endocytosis (CIE) [38]. By analyzing viability, we determined that all endocytosis inhibitors revealed a similar level of protection against MSDH toxicity (Figure 7B). Taken together, our experiments indicate that MSDH is internalized via the formation of vacuoles. The specific form of endocytosis could not be defined, as inhibitors against all studied pathways were effective. However, inhibitors targeting CME provided more protection against lysosomal membrane damage compared to CIE.

## 3. Discussion

This study shows that results from leakage experiments with liposomes challenged with MSDH can be transferred to a cell culture system to explain the damaging mechanisms. The lipid content of the plasma membrane of cultured fibroblasts is estimated to contain approximately 30% cholesterol [39], while Chinese hamster ovary (CHO) cells were found to contain 64% [40]. Moreover, cholesterol is unevenly distributed in cellular membranes. The plasma membrane of fibroblasts contains 90% of the total cellular cholesterol, leaving intracellular membranes such as lysosomes fairly empty of cholesterol [39]. Thus, to mimic the lysosomal membrane and plasma membrane, respectively, we generated liposomes composed of phospholipids only and phospholipids with 40% cholesterol, and characterized leakage of small (ANTS/DPX) and large molecules (TRITC-dextran 40 kDa).

Our findings show that the effect of MSDH on lipid membranes is highly dependent on pH. At pH 7, the results are consistent with the immediate formation of small pores, through which small fluorophores can leak. Compared to cell culture conditions, a ratio between MSDH and plasma membrane lipids below two should be used to avoid the direct lysis of the plasma membrane. As presented in Figure 4A, MSDH concentrations above 20 µM induce a loss of plasma membrane integrity and would rather kill the cell by rupturing the plasma membrane than enter the cell and promote LMP by lysosomal accumulation.

Based on previous findings [26] and the liposome experiments at pH 5, we establish that MSDH is highly charged at this pH, and that its partitioning into the membrane is kinetically impeded and thus prolongs the time required to observe the leakage of small fluorophores. However, at higher MSDH:lipid ratios, i.e., those needed to induce the leakage of large molecules, the pattern is inverted and the time required to achieve complete leakage of large molecules is shorter at pH 5 than at pH 7. Under these circumstances, the partitioning of MSDH into the lipid membranes is probably favored by its high concentration, which is why charge is no longer rate-limiting in terms of inducing leakage. In concordance, LMP is detected within 5 min of MSDH exposure and the lysosomal marker proteins, cathepsin B and NAG, are released to the cytosol. The molecular weight of cathepsin B and NAG is 31 and 140 kDa, respectively, indicating that substantial lysosomal permeabilization must occur for these proteases to be released. In the living cell, major damage is counteracted by the sequestration of the leaky organelles by lysophagy [41]. This has been shown both in vitro and in vivo, where both LLOMe and acute hyperuricemic nephropathy can activate the lysophagic clearance of lysosomes that are damaged enough to release hydrolytic enzymes [42].

Endocytic vesicles derived from the plasma membrane will fuse with lysosomes and one possibility is that LMP is the result of MSDH partitioned into the plasma membrane. However, it would require a localized high concentration of MSDH, which would probably result in plasma membrane permeabilization rather than LMP. Therefore, the selective permeabilization of the lysosomal membrane that we observe in the presented cell models is most likely the consequence of the disassembly of MSDH vesicles inside the lysosomal compartment. Considering that the area per MSDH molecule is approximately 50 A^2^ and that the radius of the vesicles formed by MSDH at pH 7 is 150 nm in average, one MSDH vesicle would consist of roughly 500,000 molecules of amphiphile. When such a vesicle is disassembled inside a lysosome due to a drop in pH, the concentration of free MSDH will range from 2 to 200 mM, considering that the diameters of lysosomes fall within 200 nm to 1 µm. Both the endocytic process and the disassembly of MSDH vesicles at low pH occurs to drive the efficient accumulation of monomeric MSDH inside the lysosomes. Since the lysosomal membrane is very low in cholesterol, the observed effect of cholesterol on the permeabilization properties of MSDH is also relevant. Interestingly, the lysosomal storage disorder Niemann Pick’s disease is caused by a mutation in the cholesterol-transporting proteins NPC1 or NPC2, which results in the accumulation of cholesterol in lysosomes. Consequently, fibroblasts from a Niemann Pick’s patient were more resilient to LMP-induced cell death [43], demonstrating that the stabilizing effect of cholesterol can be applied on the lysosomal membrane of living cells.

The Critical Aggregation Concentration (CAC) of MSDH is at approximately 10 µM [26], meaning that the pH-dependent assembly of MSDH into closed vesicles occurs at concentrations above 10 µM. Noteworthily, MSDH did not exhibit cellular toxicity at concentrations below CAC, i.e., at 5 µM (Figure 4B), indicating that vesicles, rather than monomers, of MSDH are taken up by cells. Since a previous analysis of zeta potential revealed that the vesicles were positively charged at a pH above 6.4 [26], we could conclude that both size and charge clearly promote endocytosis over free diffusion across the plasma membrane. The TEM micrographs revealed an increased number of small vacuoles (≤100 nm) close to the plasma membrane that seem to bear a protein coat. After 15 min, larger uncoated vacuoles (≈0.2 µM) were found, which, according to their size, are most likely macropinosomes [39,44]. Although lysosomal damage triggers TFEB-mediated lysosomal biogenesis [12], the immediate increase in vacuolar structures and their localization near the plasma membrane suggests that these vacuoles are formed through endocytosis rather than the reformation of lysosomes [29,45]. Using endocytosis inhibitors, we found significant reduction in lysosomal damage for all inhibitors, and the most effective were those targeting clathrin-mediated pathways. However, cell viability was rescued to the same extent, regardless of which inhibitors were used. In a recent study, Kang et al. followed the uptake of liposomes with different surface charges and found that liposomes were taken up by clathrin-mediated endocytosis in NIH/3T3 fibroblasts, irrespective of their charge [46]. The absence of highly selective pharmacological inhibitors presents a challenge when studying endocytic pathways [47]. It is still unclear exactly how many mechanisms exist, and blocking one pathway might induce the upregulation of alternative routes in a compensatory mechanism [28,48]. Even though establishing an exact mechanism proved difficult, our results clearly demonstrate the endocytic uptake of MSDH.

Cationic amphiphilic drugs have been shown to clinically enhance the efficacy of chemotherapy by accumulating in the lysosomes and inducing LMP [49]. This stresses the possibility to use lysosomotropic drugs in cancer therapy to target lysosomal stability. We conclude that the chemical property of MSDH in forming vesicles at physiological pH facilitates its endocytic uptake, and its transportation to the lysosomes. At pH 5, which is comparable to the acidic lumen of lysosomes, MSDH vesicles are disassembled into smaller aggregates that have lysosomotropic characteristics and, when reaching a sufficiently high concentration, can permeabilize the membrane. Thus, MSDH constitutes a good model for studies of the mechanisms of lysosome-dependent cell death and molecular structures with similar chemical properties. MSDH is an interesting candidates for targeted delivery to lysosomes.

## 4. Materials and Methods

### 4.1. MSDH

MSDH was synthesized according to a previously published protocol [24] and the purity was verified as described in Villamil Giraldo et al. 2016 [26].

### 4.2. Preparation of Liposomes

Lipids (dioleoyl-phosphatidylcholine (DOPC), dioleoyl-ethanolamine (DOPE) in a 60/40 ratio) were mixed in chloroform. When indicated, 40% cholesterol was included, still under a constant DOPC/DOPE ratio (all in molar ratio). The chloroform was evaporated during rotation under nitrogen to a thin film, and residual chloroform was removed by keeping the samples in a vacuum for at least 3 h. The lipid films were hydrated in 10 mM HEPES pH 7.2, 150 mM KCl, containing the corresponding fluorophore (see below) to a final lipid concentration of 5 mg/mL. The lipid suspension was subjected to five freeze-thaw cycles and unilamellar vesicles were obtained by extruding the suspension through 100 nm polycarbonate membrane filters (Nucleopore). The sizes of the lipid vesicles were confirmed by dynamic light scattering (ALV GmbH, Langen, Germany).

To prepare large unilamellar vesicles (LUVs) encapsulating the dye/quencher pair 8-aminonapthalene-1,3,6 trisulfonic acid/p-xylene-bis-pyridinium bromide (ANTS/DPX) 12.5 mM ANTS and 45 mM DPX were included in the buffer used to hydrate the lipid film. After extrusion, a Sephadex PD MidiTrap G-25 desalting column (GE Healthcare, Uppsala, Sweden) was used to remove non-encapsulated fluorophore and to exchange the buffer of the vesicle solution when required.

To prepare LUVs encapsulating TRITC-dextran 40 (tetra-methyl rhodamine isothiocyanate–dextran), 50 µM TRITC-dextran 40 was included in the buffer used to hydrate the lipid film. An Amicon filter with a 100 kDa cutoff (Sigma, Saint Louis, MO, USA) was used to remove the non-encapsulated fluorophore and a Sephadex desalting column (GE Healthcare) was used to exchange the buffer when necessary.

The final phospholipid content was estimated by measuring the phosphorus concentration according to the Bartlett method [50].

### 4.3. Fluorophore Leakage Experiments

Lipid vesicles encapsulating the dye/quencher pair ANTS/DPX were resuspended to a final lipid concentration of 50, 100 or 150 µM in 10 mM HEPES pH 7.2, 150 mM KCl or 10 mM MES (2-(N-morpholino)ethanesulfonic acid) pH 5, 150 mM KCl. In either case, the last step of the vesicle preparation consisted of passing the vesicle solution through a desalting column in which the corresponding buffer was used. Increasing concentrations of MSDH (resuspended in the corresponding buffer) were added to the vesicle solution so that the ratio of MSDH:lipids ranged from zero to eight. Leakage was assessed by measuring the emission of ANTS (λ_exc_ 380 nm, λ_em_ 520 nm) in a Cary fluorescence spectrophotometer (Varian). Experiments were performed at room temperature at ~5 min intervals. The emission obtained after adding 0.1% Triton X-100 was considered to correspond to 100% leakage and was used to normalize the values. With a CMC of 0.22 mM, 0.1% is equivalent to approximately 70× the CMC of Triton X-100. The complete disruption of the lipid vesicles under this condition was confirmed by dynamic light scattering.

### 4.4. FCS Measurements

FCS measurements were performed on a Zeiss 780 confocal microscope (Zeiss, Jena, Germany) fully equipped for FCS analysis. The sample was excited by the 514 nm laser line and focused through a C-Apochromat 40X/1.2 NA water-immersed objective via a dichroic mirror. The fluorescence was detected by the same objective and was spectrally divided and detected by a 32-channel GaAsP detector after passage through a pinhole in the image plane. By measurement using the dye Rhodamine 6G, using D_Rh6G_ = 4.14 × 10^−10^ m^2^·s^−1^ [51], the radius ω and the confocal detection volume were estimated to ω = 0.23 µm and V_dv,514_ = 0.43 fL, respectively. FCS analysis was performed using the Zen 2012 software (Zeiss, Jena, Germany) as well as in-house written functions using Origin 9.1 (Originlab Corporation, Northampton, MA, USA). When estimating the mean diffusion time in samples where free dextran molecules as well as dextran-loaded liposomes are present, the number of liposomes will be overestimated due to their higher brightness. In principle, the difference in brightness can be accounted for, but in practice this introduces large uncertainties. Mean diffusion times were therefore estimated from the half-amplitude of the curves.

### 4.5. Cells and Culture Conditions

Human fibroblasts (AG1518) from Coriell Institute (Camden, NJ, USA) were cultured in Eagle’s minimum essential medium supplemented with 10% fetal calf serum, 2 mM glutamine, 50 IU/mL penicillin-G and 50 μg/mL streptomycin (all from Gibco, Paisley, UK). Cells were incubated in humidified air with 5% CO_2_ at 37 °C and subcultured once a week. Experiments were performed between passages 12–24. Prior to experiments, fibroblasts were trypsinized and seeded at a density of 8000 cells/cm^2^ to obtain 80% confluence on the day of the experiment. When indicated, cells were pre-treated with colchincine (10 µM, 60 min), cytochalasin B (5 µM, 60 min), chloroquine (10 µM, 30 min), mono-dansylcadaverine (MDC, 50 µM, 30 min), filipin (0.5 µM, 30 min) or the vehicle (dimethyl sulfoxide (DMSO)). The medium was then changed and cells were treated with MSDH for indicated times and at indicated concentrations.

If not stated otherwise, chemicals were obtained from Sigma-Aldrich (St. Louis, MO, USA).

### 4.6. Viability and Apoptosis Analysis

Cell viability was analyzed using the 3-(4,5-dimethylthiazol-2-yl)-2,5-diphenyltetrazolium bromide (MTT) reduction assay. Cell cultures were incubated with 0.25 mg/mL MTT for 2 h at 37 °C. The MTT solution was removed and the formazan product was dissolved in DMSO. The absorbance was analyzed at 550 nm in a Victor 1420 multilabel plate reader (PerkinElmer, Waltham, MA, USA). The plasma membrane integrity was analyzed as the LDH leakage into the conditioned medium using the Pierce LDH Cytotoxicity Assay Kit according to the manufacturer’s instructions (Thermo Scientific, Waltham, MA, USA). LDH release was correlated to total LDH activity, obtained by rupturing the plasma membrane using 1X lysis buffer provided in the kit.

Caspase-3 activity was analyzed using the substrate Ac-DEVD-AMC (Becton Dickinson, Mountain View, CA, USA) according to the manufacturer’s directives. Fluorescence was analyzed in a Victor 1420 multilabel plate reader (λ_ex_ = 380 nm, λ_em_ = 460 nm, PerkinElmer, Waltham, MA, USA) and correlated to protein content, as determined by the Bio-Rad DC Protein Assay (Bio-Rad Laboratories, Hercules, CA, USA).

### 4.7. Immunocytochemistry

Cells seeded on cover slips were prepared for immunocytochemistry as described elsewhere [25]. Antibodies against galectin-1 (rabbit polyclonal, Abcam), LAMP2 (mouse monoclonal, Southern Biotech) and cathepsin B (rabbit polyclonal, Athens), followed by secondary antibodies conjugated to Alexa Fluor (Invitrogen, Carlsbad, CA, USA) were used. The specimens were mounted in ProLong gold antifade mount with DAPI (2-[4-(aminoiminomethyl) phenyl]-1*H*-Indole-6-carboximidamide hydrochloride) and examined with a Zeiss laser scanning confocal microscope using 40× objective NA 1.3 (Carl Zeiss AG, Oberkochen, Germany). Colocalization was analyzed in ≥4 randomly selected areas using the Pearson’s colocalization coefficient. To quantify punctuate galectin-1 staining, specimens were blinded and five randomly selected areas with a minimum of 20 cells in each area were quantified as either positive or negative for punctuate staining.

For live cell imaging, cells were stained with Lysotracker Red DND-99 (200 nM, 30 min; Thermo Fischer, Waltham, MA, USA) and visualized in a Zeiss laser scanning confocal microscope using 40× objective NA 1.3.

### 4.8. Cytosolic Extraction

Lysosomal release was estimated by the digitonin-mediated extraction of the cytosol. Extraction buffer (250 mM sucrose, 20 mM HEPES, 10 mM KCl, 1.5 mM MgCl_2_, 1 mM EGTA, 1 mM EDTA, 1 mM Pefablock, pH 7.5) containing digitonin (25 µg/mL for cytosolic extracts and 200 µg/mL for total protein extracts; Sigma-Aldrich, St. Louis, MO, USA) was added to cell cultures and incubated on ice under agitation. After 12 min, the extraction buffer was collected and subjected to further analysis.

### 4.9. Determination of β-N-acetyl-glucosaminidase (NAG) Activity

Lysosomal membrane stability was assayed by measuring the cytosolic presence of lysosomal proteases and comparing this to the total protease activity. Cytosolic and total protein extracts obtained by digitonin extraction were incubated with 0.65 mM substrate (4-metylumbelliferyl-2-acetamido-2-deoxy-β-D glucopyranosid, Sigma) for 40 min at 37 °C. Fluorescence was measured using a Victor 1420 multilabel plate reader (λ_ex_ = 355 nm, λ_em_ = 460 nm). For total enzyme activity, extracts containing 200 µg/mL digitonin were analyzed.

### 4.10. Western Blot

Cytosolic extracts correlated to the number of cells were prepared for immunoblot analysis as described previously [25] and probed with anti-cathepsin B (rabbit polyclonal, Athens, 1:1000) or anti-lactate dehydrogenase (rabbit polyclonal, Abcam, 1:10,000), followed by HRP (horse radish peroxidase)-conjugated goat-anti-rabbit antibody (DAKO, 1:3000). Densitometric analysis was performed using the Image Lab Software (Bio-Rad Laboratories, Hercules, CA, USA). To analyze the release of cathepsin B, the level of cathepsin B was correlated to the level of LDH for each sample.

### 4.11. TEM

Fibroblasts grown on coverslips were fixed in 2% glutaraldehyde in 0.1 M sucrose–sodium cacodylate HCl-buffer (pH 7.2) and post-fixed in osmium tetroxide. Dehydration, en bloc staining with uranyl acetate and embedding in Epon-812 were performed in the cell culture dish. Thin sections (50–70 nm) were cut with a diamond knife, stained with lead citrate and examined and photographed in a JEOL transmission electron microscope at 100 eV.

### 4.12. Statistical Analysis

Data were statistically evaluated using Student’s t-test between two groups and one-way ANOVA, followed by a multiple comparison post-test using Dunnett’s (comparison to untreated control) or Sidak’s (comparison to only MSDH) tests. The results are presented as the mean and standard deviation (SD) of ≥3 independent experiments. Differences were considered significant at *p* ≤ 0.05.

## Figures and Tables

**Table 1:** FCS decay times of TRITC-dextran 40 encapsulated liposomes.

Detergent	Liposomes pH 5 (milliseconds)		Liposomes pH 7 (milliseconds)	
	DOPC/DOPE	+Cholesterol	DOPC/DOPE	+Cholesterol
Triton X-100	0.30 ± 0.01	0.33 ± 0.01	0.28 ± 0.01	0.30 ± 0.01
MSDH:lipid 20:1	0.38 ± 0.07	0.48 ± 0.07	0.34 ± 0.04	0.51 ± 0.02*

Decay times for TRITC-dextran 40 encapsulated in liposomes with or without 40% cholesterol at pH 5 or 7 after the addition of Triton X-100 or MSDH (ratio MSDH:lipid is 20). Mean ± SD (*n* > 3), * *p* < 0.05 when comparing liposomes with and without cholesterol at pH 7.

## Abbreviations

ANTS	8-Aminonapthalene-1,3,6 trisulfonic acid
DPX	p-Xylene-bis-pyridinium bromide
FCS	Fluorescence correlation spectroscopy
LAMP2	Lysosomal-associated membrane protein-2
LD	Lysosomotropic detergent
LDH	Lactate dehydrogenase
LLOMe	L-leucyl-L-leucine methyl ester
LMP	Lysosomal membrane permeabilization
MDC	Mono-dansylcadaverine
MSDH	O-methyl-serine dodecylamine hydrochloride
MTT	3-(4,5-dimethylthiazol-2-yl)-2,5-diphenyltetrazolium bromide
NAG	β-N-acetyl-glucosaminidase
TRITC-dextran 40	Tetra-methyl rhodamine isothiocyanate–dextran with MW 40 kDa
